# The scholar as craftsman: Derek de Solla Price and the reconstruction of a medieval instrument

**DOI:** 10.1098/rsnr.2013.0062

**Published:** 2014-02-05

**Authors:** Seb Falk

**Affiliations:** Department of History and Philosophy of Science, University of Cambridge, Free School Lane, Cambridge CB2 3RH, UK

**Keywords:** Cavendish Laboratory, medieval scientific instrument, Derek de Solla Price, Lawrence Bragg, Whipple Museum of the History of Science, reconstruction

## Abstract

The Royal Society Conversaziones were biannual social evenings at which distinguished guests could learn about the latest scientific developments. The Conversazione in May 1952 featured an object that came to be called King Arthur's Table. It was a planetary equatorium, made in Cambridge's Cavendish Laboratory at the behest of Sir Lawrence Bragg. Conceived by the historian of science Derek de Solla Price as a huge, tangible realization of Chaucerian astronomy, it was displayed at the new Whipple Museum of the History of Science, discarded, stored incognito, catalogued with that whimsical name, and finally re-identified in 2012. This article examines the biography of that object and, through it, the early, inchoate years of the discipline of history of science in Cambridge. The process of disciplinary establishment involved a range of actors beyond well-known figures such as Herbert Butterfield and Joseph Needham; the roles of Price and Bragg are highlighted here. Study of these individuals, and of the collaboration that brought about the reconstruction, reveals much about the establishment of a discipline, as well as changing scholarly and curatorial attitudes towards replicas.

On the evening of 22 May 1952 Derek de Solla Price (1922–83) presented a curious object at the Royal Society's biannual Conversazione.^1^ The polished wooden disc and brass ring with revolving pointer certainly caught the attention of guests at Burlington House.^2^ Partly this was because it was associated with Geoffrey Chaucer: Price's discovery of what appeared to be a hitherto unidentified draft of a unique scientific work in Chaucer's own hand had made headlines worldwide over the previous few months,^3^ and this object, a planetary equatorium, was produced according to the instructions in the fourteenth-century manuscript.^4^ Partly, of course, attention was attracted by its sheer size: 6 feet in diameter, precisely as prescribed by the manuscript. Yet it must have appeared strangely simple next to the other exhibits that had been made in the same workshop and were displayed nearby: X-ray apparatus from Cambridge's Cavendish Laboratory.^5^ The BBC's Light Programme, which had observed the construction of the equatorium, noted the incongruity that ‘this instrument, designed more than five hundred years ago, [should] have first been made in a laboratory famous for atomic research.’^6^

How had this come about? Price was later to become famous as a historian of science and the ‘father of scientometrics’,^7^ but in 1952 he was a student, in the first year of his doctorate at Cambridge. Moreover, although history of science was a new and fast-growing subject at Cambridge, the Cavendish is not usually credited with any significant role in the subject's development; rather, that Laboratory was the epitome of cutting-edge research. It had become a household name in the days of Thomson and Rutherford; now, under Sir Lawrence Bragg (1890–1971), it was conducting pioneering work in fields such as crystallography, electron microscopy and fluid dynamics, work that would reach its apotheosis with the discovery of the structure of the DNA molecule the following year.^8^ But as well as being an exceptional manager of scientific research, Bragg had another facet, much less recognized, as a sponsor of the history and heritage of science. In this he was assisted by Price; in return, Bragg provided invaluable support at a crucial early stage in Price's career.

The equatorium was to have a long and complex life within and outside Cambridge, through its display in the University's Whipple Museum of the History of Science, removal to a storage facility, return to the museum as an unfamiliar object in the 1980s, cataloguing with the name ‘King Arthur's Table’, and eventual identification as the product of Price and Bragg's collaboration at the Cavendish, late in 2012. It is almost exactly the same age as the discipline of history of science in Cambridge, which flowered in 1951 with the opening of the Whipple Museum and the setting of the first examination paper within the University's Natural Sciences Tripos. As such, its biography will allow us to approach the historiography of science, both at Cambridge and more widely, from some new, potentially profitable angles. This object draws our attention towards important but less studied figures in the history of the discipline, such as Bragg and Rupert Hall (1920–2009); their roles in developing an institutional framework for, and curatorial attitudes towards, the history of science may be assessed through this object. In addition, there is Derek Price himself, whose career in the field is justly celebrated but has yet to be placed in its historical context. Lastly, as a physical product of the historical study of science, King Arthur's Table is part of the material culture of the field. Material culture studies are very popular at present,^9^ but so far little has been written about the role of replicas in the historiography of science.^10^ This object was not the first replica in the Whipple collection, and many have been added since, but the unique twists and turns of its biography have much to tell us about the place of replicas in museums, and the changing currents of curatorial attitudes towards them.

## Early history of science in Cambridge: historians versus scientists

The establishment of a University Department and Museum of the history of science in the decade after World War II represented the flowering of seeds planted in 1936, with an exhibition of scientific treasures in the Old Schools, masterminded by the Oxford antiquarian R. T. Gunther,^11^ and the organization of a series of public lectures on the recent history of science by a newly founded committee led by the Cambridge scientists Joseph Needham and Walter Pagel.^12^ In 1944 the Director of the Cambridge Scientific Instrument Company, Robert S. Whipple, presented a substantial collection of instruments and books to the university; it was envisaged from the start that ‘his collection would form a valuable nucleus for a Museum of the History of Science.’^13^ Difficulties in finding accommodation, the lack of a curator, and a fire in a storage unit delayed the opening of the Whipple Museum for seven years, but the subject did not stand still in the meantime. At a national level, accelerating enthusiasm led to the foundation of the British Society for the History of Science in 1947.^14^ In Cambridge a conflict for control of the subject was developing, one in which Derek Price, Rupert Hall and Lawrence Bragg would have supporting but significant roles.

In 1936 Needham and Pagel had set up a History of Science Committee, but their departure from Cambridge (Pagel to London, and Needham to China) during World War II allowed control of the committee to pass into the hands of a coterie of humanities scholars led by Herbert Butterfield. This made ‘a rather depressing impression’ on Needham when he returned in 1948: in a confidential letter to the historian of medicine and technology Charles Singer he complained:

the committee seems to have become dominated by professional historians. … They all made a great song and dance about the impossibility of history of science being done except by professional historians, which I took rather to heart, as I felt it affected my personal work … and also because I believe it to be pure nonsense.^15^

The fear of the liberal humanists in the committee, as the theologian Charles Raven wrote candidly to Needham, was the ‘real danger that the History of Science may become a convenient refuge for second rate scientists … [who] do not yet recognise that the study of history cannot be undertaken without a certain discipline and training.’^16^ Raven, it seems, saw himself as a mediating figure in this conflict: in June 1951 he wrote to Rupert Hall, honorary curator of the newly opened Whipple Museum and a fellow of Christ's College, where Raven was Master, to say ‘I do feel completely convinced the H. of S. must remain a cross-faculty effort—not, as Butterfield wants, assumed under History nor, as the scientists may easily envisage, a side-show of their own.’^17^ But Butterfield was implacable in his determination to exclude anyone who had received their primary training in the sciences from academic posts in the new discipline. As he made clear in a letter to Hall in 1956, this included Needham himself, even—or perhaps especially—after he began publishing his magisterial *Science and civilisation in China* series.^18^ For Butterfield, in the fight for true historicity scientists were clearly the enemy; he thought their histories triumphalist and present-centred. It was obviously to them that he was referring when he wrote that history of science would not fulfil its potential as a bridge between the arts and sciences ‘if we construct our story of science by drawing lines straight from one great figure to another.’^19^

This is where Rupert Hall and, as his assistant at the Whipple Museum, Derek Price came in. Because Butterfield was concerned to reconstruct the oft-ignored ‘blind alley[s]’ into which scientific development had frequently run, the physical vestiges of both scientific ‘misfires’ and ‘progress’ were invaluable.^20^ Butterfield had already shown some interest in historic scientific apparatus, setting a question on scientific instruments in the seventeenth century in the Modern History examination,^21^ and it is not surprising that under his direction the History of Science Committee was keen to take on responsibility for the Whipple collection, as well as the many instruments scattered around various departments and colleges.^22^ Marking the Whipple Museum's opening in 1951, Hall, a historian first and foremost whose appointment as lecturer and later curator was actively encouraged by Butterfield,^23^ wrote:

the instruments and books offer a remarkably full conspectus of the history of science since the Renaissance, and a useful reminder that besides the drama of the revolutions in thought effected by a Newton, a Lavoisier or a Pasteur, it must not neglect the slow evolution of instruments, education and public understanding through which the present prestige of science has arisen.^24^

This approach, reminiscent of Gunther's description of instruments as ‘milestones in the history of English science’,^25^ chimed with the local emphasis of Butterfield and his fellow liberal humanists.^26^ Conversely, it was anathema to the Marxist tendencies of Needham, who with Walter Pagel, in their edition of the 1936 lectures, had lamented, ‘historians of science have tended too much to fall into mere antiquarianism.’^27^ However, as we shall see, antiquarianism won out at the Whipple: the priority, much as had been stated in the 1944 memorandum proposing the creation of the museum, was ‘to portray the outstanding discoveries made in Cambridge during the present century’; to bring together ‘apparatus [that] may be entirely lost or destroyed unless early provision is made for its permanent preservation.’^28^

## Derek J. Price: scholar and celebrity

It was into this environment that Derek J. Price arrived in the winter of 1950–51. Because his background, personality and relations with his colleagues are crucial to the subject of this article, some biographical details are useful here.^29^ Born into a working-class family in 1922, at the age of 16 years he began working as a laboratory assistant at the newly established South West Essex Technical College.^30^ Under the supervision of the college principal, Harry Lowery, he progressed to a first-class honours BSc in 1942 and a PhD in 1946; his research at that time was on the infrared emissivity of metals at high temperatures.^31^ After spending 1946–47 at Princeton University with a Commonwealth Fund Fellowship, he took a post teaching applied mathematics at the University of Malaya.^32^ It is unclear when his interest in the history of science first developed, but it clearly consumed much of his free time in Singapore.^33^ It was while there that he had the experience of stacking a complete set of *Philosophical Transactions of the Royal Society* by decades, and witnessing the ‘fine exponential curve’ they formed against his study wall.^34^ (The mythical quality that this episode has acquired in the historiography of science owes much to Price's considerable gifts of self-promotion.)

His mind made up to pursue the history of science further, Price proceeded purposefully. Despite having a wife and baby daughter to support, he gave up his post in Singapore and moved to Cambridge, hoping to secure a research fellowship.^35^ The first person with whom he made contact was Lawrence Bragg. The two had met when Bragg was on the selection committee for the Commonwealth Fund Fellowship;^36^ Bragg, who had recently acquired an interest in the historical apparatus of the Cavendish Laboratory, quickly realized that Price was ideally qualified to catalogue the Laboratory's objects and correspondence.^37^ Price also met with Butterfield, who adjudged him ‘a very plausible kind of person’ and introduced him to Rupert Hall, who was similarly impressed.^38^ It seems that Price quickly came to accept that he would have to enrol as a doctoral student at Cambridge; with encouragement from Butterfield and Hall, he applied to join the Faculty of History, and Christ's College, for admission in the Easter Term of 1951 with the research title ‘The history of scientific instrument making’.^39^ The references that he supplied with his Cambridge application were uniformly glowing. The maritime historian Cyril Northcote Parkinson praised his ‘first-class brain, exceptional energy and willingness to make great sacrifices … to enter his chosen field of study’,^40^ his vice-chancellor T. H. Silcock highlighted his ‘intelligence and originality’,^41^ and his old supervisor Harry Lowery called him ‘a brilliant research worker, extremely keen on his work. He is full of ideas and is a very good experimenter, being able to circumvent difficulties when he cannot solve them directly.’^42^ He added that Price ‘has a genial disposition and gets on well with people.’

Price's experiences in Cambridge and thereafter would corroborate everything that his referees wrote in 1951 about his ability and appetite for hard work. However, not everyone who came into contact with him shared Lowery's opinion of his character. Even before Price came to Cambridge, Bragg suspected, from what he had heard of Price's time at Princeton, that Price was ‘rather changeable’.^43^ Better acquaintance with Price over the succeeding years did not fully erase his doubts: while recommending Price for the post of Assistant Keeper at the National Maritime Museum in 1959, he expressed the reservation that ‘Price is a man of single-minded purpose and in pursuing his aims with such tenacity and keenness he may sometimes tread on people's toes.’^44^ In more personal correspondence, he quoted his wife's epithet: ‘not socially house-trained’.^45^ Other members of the scientific establishment agreed. Charles Singer, for example, while frequently using the word ‘genius’ in connection with Price, also noted, ‘I don't think he has quite learnt to handle people.’^46^ In Bragg's estimation ‘there is nothing wrong with the man himself, it is his background.’^47^ This last comment raises the possibility that Price was the victim of snobbery by those in Cambridge who objected to his working-class, technical-college background.^48^ It is also possible that his Jewish roots were a factor,^49^ although it does not seem that he made these public and, in any case, many people of Jewish origins had been successful in Cambridge by that time. The person at Cambridge who knew Price best—his doctoral supervisor and boss at the Whipple Museum, Rupert Hall—is the one who was most reticent in his criticism; but Hall's correspondence strongly suggests that their relationship was cool, and implies that he had a low opinion of Price's standards of scholarship.^50^ For example, when in 1956 Price was preparing to move to the USA, Hall wrote to his friend (and future wife) Marie Boas, sarcastically wishing her ‘good luck with the Prices. They will be with you soon: aren't you lucky?’^51^ She responded, ‘don't expect you can dump your mass produced cheap wares over here; we've got protective tariffs.’^52^ Bragg expressed the view that Hall could have done more to support Price's career.^53^

Price himself was aware that the way he went about his work could upset people.^54^ And his refusal to pander to the polite conventions of academic etiquette was never more obvious than in his research into Peterhouse MS 75, leading to the construction of the equatorium at the Cavendish Laboratory. Having been accepted by the Faculty of History as from April 1951, Price's short-term future at Cambridge was secured when he was selected for an Imperial Chemical Industries (ICI) Fellowship at the beginning of June. This was despite the ICI Fellowships' being intended for original research of a directly scientific nature;^55^ in the ‘long discussion’ noted in the Fellowship committee minutes, the support of Lawrence Bragg as one of the nine managers was surely crucial to Price's success.^56^ Although the Fellowship was only for one year (it was subsequently renewed for a further two),^57^ the £600 stipend was a lifeline for Price and his growing family.^58^ He had already begun his research, combing through manuscripts in the various university and college libraries of Cambridge, but now pursued it with renewed vigour. The breakthrough came after six months (during which he had also begun working part-time in the new Whipple Museum),^59^ when Price came to examine a manuscript in the Peterhouse library. Gunther had identified this as just another fourteenth-century astrolabe treatise,^60^ but Price quickly realized it was something different:

It was a rather dull volume, traditionally attributed to an obscure astronomer, and it had probably hardly been opened in the last five hundred years it had been in the library.

As I opened it, the shock was considerable. The instrument pictured there was quite unlike an astrolabe—or anything else immediately recognizable. The manuscript itself was beautifully clear and legible, although full of erasures and corrections exactly like an author's draft after polishing (which indeed it almost certainly is) and, above all, nearly every page was dated 1392 and written in Middle English instead of Latin. …

The significance of the date was this: the most important medieval text on an instrument, Chaucer's well-known *Treatise on the Astrolabe*, was written in 1391. To find another English instrument tract dated in the following year was like asking ‘What happened at Hastings in 1067?’ The conclusion was inescapable that this text must have had something to do with Chaucer. It was an exciting chase.^61^

**Figure 1.**
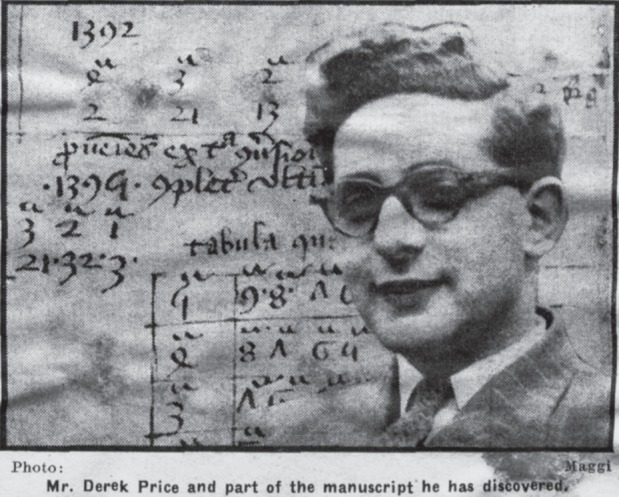
Image of Derek Price and Peterhouse MS 75.1, published in *Varsity* on 23 February 1952.

This account was published almost a decade later, but it encapsulates the breathless flair of much of his writing on this subject. Price knew how to tell a story, and he did not wait long after his discovery in December 1951 to begin doing so. By the end of February 1952, unchecked by the birth of his second child (named Jeffrey, though he claimed that the similarity to Chaucer's name was coincidental^62^) in January, articles on the subject had been published in *The Times* and the Cambridge University newspaper *Varsity* (figure 1);^63^ a detailed two-part account in *The Times Literary Supplement*, and worldwide publicity, followed shortly thereafter.^64^ Just a couple of weeks after the discovery, Price was able to inform Robert Whipple (and Whipple relayed to Hall), ‘the Univ. Press is going to publish his account of the manuscript in a special book. This is, indeed, a triumph!’^65^ And Price's triumph was sealed when, the manuscript having been disbound, a word that had previously been partly concealed was revealed as ‘Chaucer’—a discovery that, according to Price, led to ‘four people (including two distinguished professors) being ejected politely [from the University Library's Anderson Room] for whooping with delight.’^66^ Convinced of the significance of his discovery, he began to make arrangements to speak about it at the Royal Society that spring. And he contacted Sir Lawrence Bragg at the Cavendish Laboratory in order to arrange first the use of the Laboratory's infrared and ultraviolet photographic equipment to analyse the manuscript,^67^ and then the construction of what would be the *coup de théâtre* at the Royal Society: a full-scale model of Chaucer's equatorium.^68^

## Sir Lawrence Bragg's Cavendish Laboratory: birthplace of a Chaucerian equatorium

Strange as it may seem, after 14 years of Sir Lawrence Bragg's stewardship the Cavendish Laboratory was ideally suited to the construction project. Opened in 1874, the Cavendish was by this time firmly established within the Cambridge landscape, and its public prestige as the locus of a stream of scientific discoveries was settled.^69^ When Ernest Rutherford died unexpectedly in 1937, Lawrence Bragg was not the automatic choice to become the fifth Cavendish Professor. Although he had been educated at Cambridge, had won a Nobel prize aged just 25 years, and had succeeded Rutherford in the professorship at Manchester, he was not a nuclear physicist in the tradition of Rutherford and his predecessor J. J. Thomson. His appointment at the age of 48 years in March 1938 was thus upsetting to many in Cambridge, as well as to his own father, who had not been happy there.^70^ The biochemist John Kendrew observed, ‘everybody thought it was absolutely terrible, the great days of the Cavendish had ended, that they had appointed this man who knew nothing about the main subject the Cavendish did, the worst appointment in the whole history of the place.’^71^ In fact, of course, it was an inspired appointment: Bragg first kept the ship steady during World War II, then performed a sweeping reorganization that allowed the Laboratory to continue at the forefront of research in the physical sciences.

Bragg had a clear vision for how research should be conducted and applied, and he combined this with an open and supportive management style. He realized that the Cavendish could not outmuscle the USA in large-scale research, not only because of financial constraints but also because of the independent traditions and decentralized structure of Cambridge University. What he could do, however, was create the conditions for innovation. Brian Pippard, himself later Cavendish Professor, ascribed to Bragg ‘great credit for creating an environment in which a multitude of ideas could prosper, and for his enthusiastic support … of every promising venture, whether or not it was directed at obviously fundamental problems.’^72^ Bragg had laid out the blueprint for his reformed Cavendish Laboratory in a lecture at the Royal Institution in 1942.^73^ He argued that the quality of the work being done in fundamental physics was as high as could be expected, but that the same could not be said of applied physics. The remedy was for physicists to spend time in industry, ideally between school and university. Although research should never be directed by industrial needs—and Bragg was particularly against the practice of collecting physicists in research institutions, away from the responsibilities of teaching but also away from the fresh ideas of young students—it should always have an eye for potential applications. He was very clear about his ideal research unit:

six to twelve scientific men and a few assistants, together with one or more first-class mechanics and a workshop in which the general run of apparatus can be constructed. … It is not wasteful to duplicate lathes and other machines by giving each group its own workshop; the time of researchers is far more expensive than the overheads on machinery.^74^

Immediately after the war, Bragg began to put this plan into practice, dividing the Laboratory into six autonomous groups, and further subdivisions. The reorganization progressed fairly smoothly but was hampered by the rapid expansion of the Cavendish from 40–45 researchers before the war to 160 by 1948.^75^ The consequent lack of space was eventually to lead to the Laboratory's relocation to larger premises in the early 1970s, but it was limited even during Bragg's professorship; partition walls had to be installed in several laboratories to create new, smaller workspaces.^76^

One casualty of this reorganization was the Laboratory archive. Crowther has bemoaned the fact that ‘the Cavendish, like the whole of British science, was sublimely disinterested in its historical aspects’, and noted that the members of the Laboratory saw the creation of a museum as a waste of money.^77^ It is true that the Cavendish archives are deficient for several areas and periods, but Bragg certainly showed an interest in the institution's heritage. Before Price came to Cambridge, Bragg had already done some research into the Laboratory's collection of historic equipment.^78^ However, he had not addressed the archives because, as he told the *Daily Telegraph*, ‘no one with the combined scientific and literary knowledge has been available.’^79^ Price's arrival changed that.^80^ Price was assiduous in collecting and cataloguing the correspondence of Maxwell and Rutherford^81^ and ‘unearthed many treasures’ in the process, to Bragg's evident delight.^82^ Price also wrote the first guide to the exhibits of the Laboratory museum.^83^ There is no evidence that he was paid for any of this work (indeed, if he had been, it would probably have been deducted from his ICI stipend).^84^ But he certainly enjoyed it, even keeping his steward's badge as a souvenir when he emigrated to the USA in 1957.^85^ And the cordial personal relationship that was cemented between Price and Bragg, as well as the knowledge that Price gained of the Cavendish and its well-equipped workshops, came in useful when he discovered the ‘Chaucer’ manuscript and had the idea of using the latest scientific technology not only to analyse it but also to follow its instructions.

**Figure 2.**
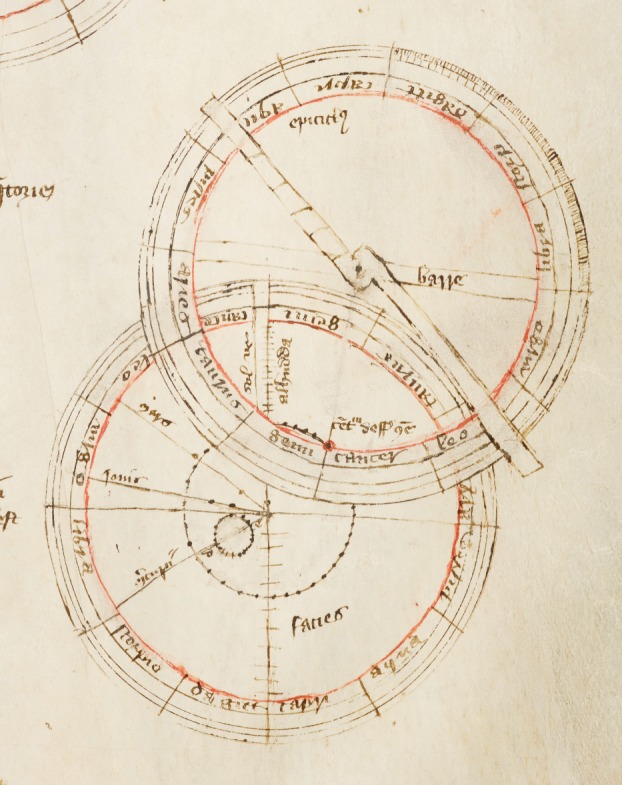
Detail from Peterhouse MS 75.1. (By kind permission of the Master and Fellows of Peterhouse, Cambridge.) (Online version in colour.)

**Figure 3.**
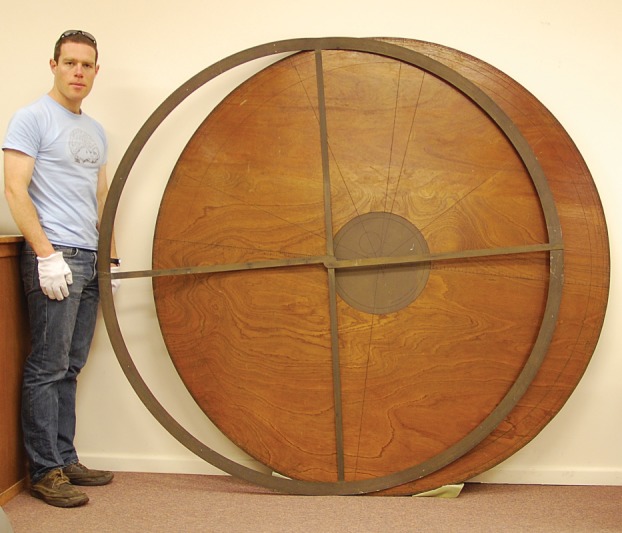
Wh.3271 (diameter 1870 mm): equatorium built at the Cavendish Laboratory for Derek Price. (Courtesy of the Whipple Museum of the History of Science, Cambridge.) (Online version in colour.)

Unfortunately the Cavendish technicians did not keep records of jobs of this nature,^86^ and Price himself did not record any details of the manufacturing process, except that ‘even with the resources and the technical staff of the Cavendish Laboratory it took many full days of work to make the full-size device properly.’^87^ It is clear, though, that Price was determined to follow every detail of the instructions in the manuscript (figure 2). Although modern machine techniques were obviously employed in its manufacture, the materials used were as close to authentic as possible. For example, machine-rolled rather than hand-hammered brass was used for the instrument's epicycle and label, but the brass is used exactly where specified by the author. The only exception to this is the ‘limb’, analogous to the rim of an astrolabe, which should be made of brass or parchment and form a circle covering the outermost two inches of the face of the equatorium.^88^ Price chose to ignore this, instead making the limb's markings of signs, degrees and minutes directly onto the wooden face. There is just one small mistake: Mercury's deferent and equant centres have been incorrectly labelled. But in all other respects the equatorium was made to a high standard, exactly as its fourteenth-century inventor specified (figure 3).

This is not a working model built to enable Price and others to understand how the instrument represented and simplified Ptolemaic theories; it is a historical reconstruction, conceived as an attractive representation of what the author of the manuscript might have imagined. So when engraving the apogee line for each planet on the face of the model, Price chose to use fourteenth-century values, rather than updating them to make the instrument more easily useable in his own day. The size of the model is its most striking feature. Price was probably already aware that the 6-foot diameter specified in the manuscript was somewhat idealistic at best, and that if this equatorium had ever been built in the fourteenth century, it would almost certainly have been considerably smaller. The fact that he went ahead with the full-size reconstruction, authentic in the sense of following the manuscript but not in terms of its faithfulness to what most probably would have been made in the later Middle Ages, makes his priorities clear. It certainly did not prevent him from learning from the construction: the bulk of the finished equatorium and the flimsiness of its brass epicycle surely confirmed his suspicion that ‘it must be considered doubtful whether large instruments were ever made in metal during the Middle Ages.’^89^ And the experience was to influence his later work, as is clearly illustrated by his contribution to Singer's monumental *History of technology*. For example, his remark (which appears in the volume edited by Hall) ‘an instrument must certainly be very large, carefully and closely divided, perfectly jointed, and made quite stable in order to secure the required accuracy’ was clearly informed by the experience of having built one of these instruments himself.^90^ Nevertheless, he did not mention the replica in his PhD thesis, despite explaining several investigatory techniques he had employed. Thus the replica was not intended primarily as a tool of historical research; rather, it was a theatrical prop for this accomplished showman.

## ‘Ancient and modern’:^91^ the Royal Society Conversazione

The show at which it was to be unveiled was a Conversazione at the Royal Society (then located in Burlington House on Piccadilly) on 22 May 1952. By this time the tradition of twice-yearly ‘social evenings when the Society seeks to promote scientific research not only by encouraging scientists to exhibit and discuss new developments, but also by entertaining’ a range of distinguished guests, had been established for a little over 100 years.^92^ It had become customary to hold one Conversazione in May and another in June, unless a special celebration or commemoration prompted one of these to be moved. At each event, around 20 or 30 individuals or groups presented their research to upwards of 500 guests; many exhibitors would return on the morning after the May Conversazione to repeat their presentations for visiting schoolchildren. The exhibits at the May and June events were mostly the same, although some would not be repeated and others might be added in their place. They tended to present current scientific research; after World War II, scientists working in industry were increasingly visible alongside those from university and research laboratory settings.^93^

The Cavendish Laboratory was invariably well represented at these events. Not only was it a leading research institution, but in Lawrence Bragg it also benefited from a Director who was well connected and who understood the importance both of publicizing the work of the Laboratory and of promoting wider understanding of science.^94^ For example, on the same evening as Price's exhibit was shown, two other groups from the Cavendish exhibited new uses of X-rays, one in metal crystallography and the other in biological microradiography;^95^ Anthony Kelly, who was part of the former group, recalls that it was Bragg who suggested they participate in the Conversazione.^96^ The historical nature of Price's research was a potential obstruction: although Conversaziones usually featured one or two historical exhibits, these invariably had some connection with the Royal Society.^97^ However, as Hall put it, ‘through Bragg's means’ the way was smoothed and Price was able to present his research at this prestigious venue.^98^

A note from the Laboratory's General Secretary, E. H. K. Dibden, to Hall shows that the Cavendish took care of the non-trivial task of transporting the bulky equatorium from Cambridge to London: the Chemical Laboratory van was booked for this purpose.^99^ Hall and Price both went,^100^ and stayed for the Schools Exhibition the following morning.^101^ As well as the equatorium, they took with them the newly rebound Peterhouse manuscript, and an object that Price believed to be ‘the only medieval equatorium still extant’,^102^ borrowed for the occasion from the Library of Merton College, Oxford.^103^ As Price himself recognized,^104^ there were several significant differences between the Merton instrument and the Peterhouse equatorium, which lead one to question why he went to the trouble of obtaining the former for his exhibit. First, although the Merton instrument's incomplete state makes it hard to be certain, it seems quite likely that the simplifications that allow the separate Ptolemaic models for planetary motion to be represented on a single instrument have been carried out quite differently from the Peterhouse equatorium; the two instruments would simply not have worked in quite the same way. Second, the Merton instrument was made for use at Oxford, whereas the Peterhouse manuscript is clearly linked to London. Most fundamental, however, is the fact that the Merton equatorium is engraved on the back of a 14-inch astrolabe: in size, materials and basic conception it is quite different from the instrument described in the Peterhouse manuscript. In his *Early science in Oxford* Gunther described it under the heading ‘Astrolabe, plumb level, and quadrant’.^105^

In contrast, Gunther's description, alongside other astrolabes at Merton and Oriel Colleges, makes explicit links with the Merton astronomer Simon Bredon—previously identified as the author of the Peterhouse manuscript—as well as implicit ones to Chaucer.^106^ Price, although dismissive of the case for Bredon's authorship of the manuscript, accepted Gunther's suggestion that the Merton instrument might be the *astrolabium maius* left to that college by Bredon in 1372.^107^ Bredon was thought by some scholars to have had links to Chaucer, and perhaps to have taught him.^108^ Thus a connection between the Merton instrument and the Peterhouse manuscript provided a further link between the manuscript and Chaucer; this could not be called evidence in support of Chaucer's authorship, but it perhaps helped to place the manuscript more firmly into the world of what Price called ‘the great school of astronomer-physicians at Merton College’.^109^ At any rate, Price was convinced enough by the connection to use a photograph of the Merton instrument as the frontispiece to his edition of the manuscript, which doubled as his PhD thesis. Similarly, bringing the Merton instrument to the Royal Society not only lent Price's exhibit greater visual appeal (something encouraged at the Conversaziones); the fact that it was a genuine medieval object, rather than a modern reproduction, surely added to the credibility of his presentation. Just as Sir William Osler, the motive force behind the 1919 exhibition of scientific relics—which certainly included the Merton instrument—catalogued in *Early science in Oxford*, had sought to clothe the New Science in the reassuring garb of the Old Humanities,^110^ so Price was appropriating some Oxonian prestige for his own, ultimately scientistic, purposes.

## Scholars/craftsmen? Derek Price and Rupert Hall

Price's presentation of the equatorium at the Royal Society was well received, meeting with favourable coverage from the mainstream media and specialist journals alike.^111^ Price presented his developing research again later that year, in a Friday Evening Discourse at the Royal Institution.^112^ Bragg, who was himself non-residential Professor of Natural Philosophy at the Royal Institution, again helped to bring this about.^113^ Rupert Hall was not present on that occasion; however, Robert Whipple was, and wrote to Hall the following day that it was ‘a great success and a finished performance’ that ‘met with great approval’ from the ‘enthusiastic audience’.^114^ It seems from Whipple's letter that Hall was unwell at that time, so his absence should not be read as a snub to Price; and in general there is no evidence that the personal differences described above affected their professional relationship. It is true that, in Price's first-year evaluation, Hall recommended that the History Faculty Degree Committee consider replacing him as Price's supervisor, but this was in light of Price's desire to change the topic of his research from ‘The history of scientific instrument making’ to ‘An edition of MS 75 (i) in Peterhouse Library’.^115^ In the event, after Price wrote to the Secretary of the Board of Research Studies threatening to withdraw from the PhD, his title change was approved; Hall continued as his supervisor^116^ and indeed was to be one of Price's PhD examiners after it proved impossible to obtain the services of Lynn Thorndike, Price's first choice.^117^ Despite the title change, and after another lengthy discussion in which Bragg once again fought his corner, Price's ICI Fellowship was extended for a further two years, with an increased stipend of £750 per year.^118^ The managers also approved Price's part-time post as assistant to Hall in the Whipple Museum.^119^

In these early years at least, Hall was ‘a continuous source of inspiration and fresh ideas’ for Price.^120^ However, it is unclear how much he influenced Price's historiographical outlook. The greatest influence on their attitudes to scientific instruments in this period was to be the work of Maurice Daumas,^121^ who benefited from energetic correspondence with the Whipple in the early 1950s and who thanked Hall, Price and another young museum assistant, David Dewhirst, in the introduction to his *Les instruments scientifiques*.^122^ Daumas set himself against the trend of decontexualized catalogues of instruments that, he noted, ‘a pour effet de ne pas rendre un compte très exact des circonstances assez complexes dans lesquelles ont été acquises … les connaissances nouvelles’, but this criticism was only published in the autumn of 1953.^123^ Before then, Gunther's antiquarian approach reigned unchallenged, which was understandable when the priority for the curators of the new Whipple Museum was to organize its contents and to begin to understand the great treasures hidden elsewhere in Cambridge.^124^ In the preface to his edition of the *Equatorie* manuscript, written in July 1953, Price noted the influence of three scholars on his work: Gunther, Thorndike and George Sarton. The reference to Sarton stands out here as a possible point of contrast with Hall, for although Sarton's encyclopaedic scope was much admired at this time, Hall found his positivistic approach rather dull.^125^ By contrast, Hall was drawn to the work of Alexandre Koyré, especially in his focus on the development of theoretical understanding. Koyré's influence is readily apparent in Hall's 1957 paper ‘The scholar and the craftsman in the Scientific Revolution’, in which, accepting the dichotomy set up by that title, he argued that whereas craft was necessarily empirical, scholarship was not; scholars drew freely on problems raised by craftsmen, but rarely addressed ‘the world in its crudest, least philosophical and most craftsmanlike sense’.^126^ Thus for Hall, as for Herbert Butterfield, science was driven by the development of ideas, rather than by great men;^127^ in their discussions of the Cambridge History of Science course, Koyré's *Études galiléennes* comes high on a very short list of ‘specially recommended monographs’.^128^ Hall was certainly no Butterfield clone—for example, the latter was always sceptical of critical editions of documents,^129^ whereas Hall is noted for his work on the correspondence of Henry Oldenburg and Isaac Newton^130^—but it is easy to see why he described Butterfield as his ‘mentor’.^131^

Price was different. It would be too simplistic to describe him as a proxy for Joseph Needham and the scientists in a conflict with Butterfield and the humanists, but he was clearly no narrow textual scholar. Hall was to define the scholar-craftsman dichotomy as quadruple, and one of its four criteria was ‘teleological’, distinguishing ‘those who seek mainly practical success through science’ from ‘those who seek mainly understanding’.^132^ The Equatorie did not confer new understanding—although equatoria in other forms could be used for educational ends—and did have a practical purpose, but it was the product of an indisputably scholarly enterprise. So Price's work on a piece of medieval scholarly technology was at the dividing line of science and craft. And Price himself, who had started as a laboratory technician and had then become an applied physicist before entering the world of liberal humanism, accumulating the requisite expertise in areas such as palaeography and codicology as best he could along the way,^133^ was already both craftsman and scholar; studying, editing and translating a medieval manuscript, and reconstructing a piece of medieval technology, were both entirely natural to him.

## King Arthur's Table: the life of a replica at the Whipple Museum

Such historiographical questions do not seem to have been a priority for Hall and Price in the early days of the Whipple Museum. Far more pressing problems were at hand. Frank Sherwood Taylor, who had succeeded Gunther as curator of the Museum of the History of Science in Oxford, argued in 1949 that the first priority should be collection and preservation; he clearly had the cultural devastation of two world wars in mind when he wrote that ‘future generations may well investigate more accurately, display more brilliantly, teach far better—but they will almost certainly be less well able to collect.’^134^ Thus Hall and Price strove to ‘ferret out’ worthy objects, whether loaned from college collections, purchased in Portobello Road, or donated by subsequent benefactors.^135^ There was no stated acquisitions policy, but Hall was deliberately catholic in his collecting, widening the scope of the original collection in both time and topic; on the other hand, many of the new arrivals were in some way linked with Cambridge. The main restriction, of course, was space. The original museum premises, at 14 Corn Exchange Street, had always been envisaged as a temporary home;^136^ the possible expansion of that site, or movement to a new, more spacious, location, was frequently discussed in the History of Science Committee.^137^ But despite various places being considered, from terraced houses to an underused city-centre church,^138^ it was not until 1959 that the museum was able to move into more suitable quarters, vacated by the Physical Chemistry Laboratory; these included the fine seventeenth-century hall of the old Perse School. The lack of space was a source of great frustration to Hall, who wrote in 1954: ‘it is something of a scandal that the Whipple benefaction should have remained for ten years in a depressed condition.’^139^ He noted a further problem quite succinctly: ‘a museum deserves a curator.’ It was not until 1969, after the Whipple family threatened legal action,^140^ that a full-time curatorship was created;^141^ before then, first Hall and then his successor, Gerd Buchdahl—a philosopher of science with no curatorial experience—were expected to fulfil the role, unpaid, ‘in a very little leisure time’.^142^

The Whipple Museum's expanding collection, restricted space and curatorship that was limited in both time and expertise inhibited the care that could be taken of the collection, as well as the quality of its cataloguing. The story of King Arthur's Table exemplifies this. The lack of systematic paperwork from the museum's early years makes it impossible to know exactly when it was removed from its prominent position on the end wall of the larger of the museum's two rooms, but what is more certain is that it left the museum site entirely when parts of the building that had been storerooms were converted into office space for Buchdahl and Michael Hoskin.^143^ Thus, when David Bryden arrived from the Royal Scottish Museum as the Whipple's first professional curator in 1970, he found that the equatorium, along with a great many other objects, were being stored in a semi-derelict building belonging to the University's Estates Department, in Thompson's Lane near the river Cam.^144^ Buchdahl had employed a junior curator from the Science Museum to list the objects, but this had been done very badly, so the resulting records were useless. Thus one of Bryden's first tasks as curator was to bring these objects back to the main Whipple site, where fresh storage space had been created by the disbanding of the Department of Colloid Science.^145^ However, Price's equatorium was not among the returning objects. The dilapidated state of the Thompson's Lane building meant that removal contractors were unwilling to use the staircase to the first-floor storeroom; to transport the collection Bryden had to use his own ‘relatively small estate car’, into which the equatorium simply would not fit.^146^ Only in 1985 was it formally accessioned into the Whipple Museum, with the simple description ‘wooden circle and brass limb divided by zodiac’.^147^ The uncertainty over its origins was such that, when the museum implemented a new electronic catalogue in the late 1990s, for the object name the cataloguer used the nickname it had acquired: ‘King Arthur's Table’.^148^ The equatorium was only re-identified in October 2012.^149^

What does this story tell us about the priorities of the museum's early curators? Alongside collection and preservation, they also had to consider the competing concerns of investigation, teaching and display.^150^ In his catalogue-cum-history *Early science in Cambridge*, Gunther had emphasized the ‘rarity’ of the objects displayed at the 1936 Old Schools exhibition, as well as their ‘association with the great men of science of other days’;^151^ likewise, at the opening of the Whipple Museum, Hall, as well as acknowledging the ‘importance’ of the benefaction, stressed ‘the variety and beauty of the work of the craftsmen who, in London, Paris, Augsburg, Nuremburg and elsewhere, have produced the tools of arts and sciences.’^152^ Although, as we have seen, treasures produced in Cambridge were by no means shunned, modern replicas might well be. Bryden suggests that he was less concerned about the equatorium's failure to fit in his car because it ‘looked modern to me’;^153^ the early collection principles focused on antique objects. Replicas did enter the museum in its first few years, but they were few: a selection of Leeuwenhoek and Musschenbroek microscopes, made by John Mayall in the 1880s,^154^ and a copy of Newton's 1671 reflecting telescope, made for Trinity College in 1953.^155^ The five Mayall microscopes were arguably of historical significance in their own right; more importantly, they were part of the original Whipple collection.^156^ They were also visually attractive and, crucially, small, requiring little storage space. The Newtonian telescope, at 9 inches, was also fairly compact and, in terms of association with great men of science, one could hardly ask for more. Because the Royal Society was unlikely to part with the original object, an exact copy must have seemed an acceptable substitute. Acceptance of the equatorium was, however, not so straightforward. Its association with Chaucer, whose ‘great man of science’ status was in any case a little problematic, was unproven; even if that association were accepted, it was the text in Chaucer's hand, rather than any physical instrument, that was of value. Even its value as a replica was not beyond question because, as already discussed, it was hard to argue that the instructions in the Peterhouse manuscript would or could have been followed literally in the fourteenth century.^157^ It could be called striking, but was not exactly beautiful. And given the space constraints within which the early curators were working, a 6-foot disc and ring (which really required a space about 10 feet wide to be displayed in their proper arrangement) were always likely to be candidates for storage.

## Conclusion

**Figure 4.**
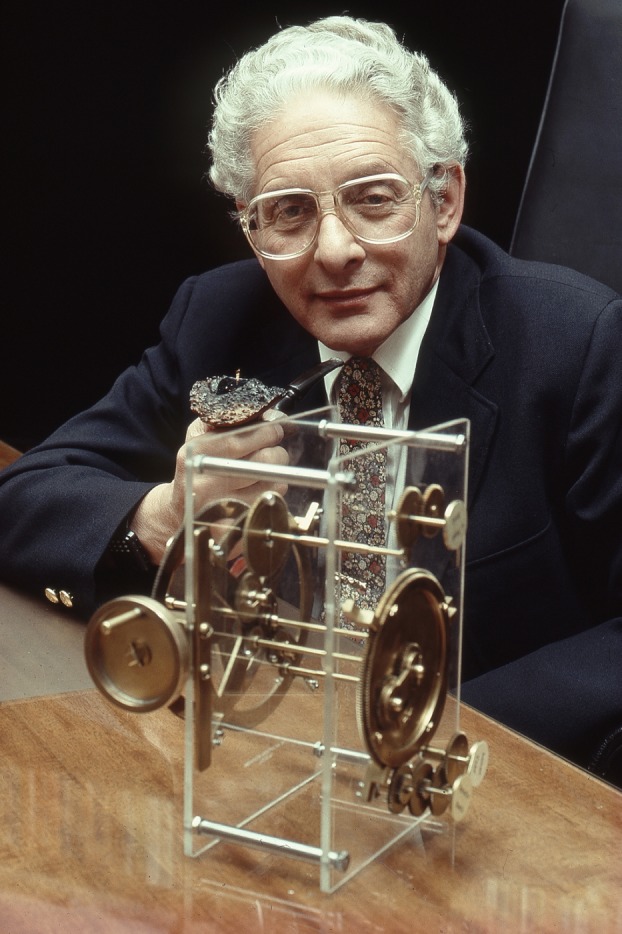
Derek de Solla Price with a model of the ‘Antikythera Mechanism’, August 1982. (Courtesy of the Price family.) (Online version in colour.)

Derek Price left the Whipple even earlier than his equatorium, departing at the end of 1956 for the USA. He held posts at the Smithsonian Institution and Princeton before moving to Yale University in 1959,^158^ where he opened a new Department of History of Science and became the first holder of the Avalon Professorship. He did not abandon his taste for showmanship and crafted a public profile for himself: a plain-speaking historian of technology who was fully at home with his subject matter; both scholar and craftsman. Photographed for *Omni* magazine by Malcolm Kirk in 1982,^159^ he posed (figure 4) with a model of the ‘Antikythera Mechanism’ as ‘the scientific detective who, after years of intermittent but concentrated study, solved the puzzle of the mysterious mechanism.’^160^ As with the Cavendish equatorium, the ‘invisible technician’ who had actually made the model was not named.^161^ But Price did not forget those who had helped him: in the Preface to *Science since Babylon*, published in 1961, he acknowledged his debts to Parkinson, Lowery, Christ's College and ‘Sir Lawrence Bragg, whose kindness and hospitality meant so much in the Cavendish Laboratory.’^162^ It is clear that his experiences in the Cavendish had a long-term effect on his work, both in terms of the way he viewed technology, and his self-image as a scholar.

Bragg, too, valued Price; they maintained a cordial correspondence for many years. Beyond the professional assistance they had given each other, it is not too much to say that Bragg learnt something from Price about the history of science, and perhaps about the promotion of science to the public. Despite his misgivings about playing the role of ‘elder statesman of science’, expected of him as Cavendish Professor and later Director of the Davy–Faraday Laboratory at the Royal Institution,^163^ he took that role increasingly seriously. He was, as we have seen, energetic in publicizing the work of his Laboratory at events such as Conversaziones and Royal Institution Friday Evening Discourses. Writing the foreword to James Watson's *The double helix*, he noted the controversy surrounding the book, and explained that he had supported its publication because of its importance as ‘an autobiographical contribution to the history which will someday be written.’^164^ In the 2013 ‘Bragg Centenary’ commemorations, attention understandably focused on his collaboration with his father in the development of X-ray crystallography.^165^ However, as this paper has showed, we should not forget his significant, if indirect, contribution to the history of science.

Bragg and Price are two among many individuals who had important roles in the development of this discipline. Butterfield and Needham, perhaps the most central figures, have been considered at length elsewhere; so, to some extent, has Hall, the ‘Cambridge-built’ Butterfield protégé whom Singer backhandedly dismissed, writing that ‘in thirty years’ time … Hall will remain what he is now, a first class and reliable teacher and writer.'^166^ The context of that statement was a comparison with Hall's doctoral student, who, Singer predicted, ‘will have passed into a commanding position of authority’ in the field. Derek Price was indeed destined for greatness, and many of his contributions to the history and sociology of science and technology have been discussed elsewhere. But the complex legacy of his achievements in Cambridge, and the impact of his sometimes topsy-turvy relationships with his superiors and colleagues, have deserved fuller consideration.

That such consideration has arisen from the biography of an object in the Whipple Museum of the History of Science is entirely appropriate, because from the very beginning the collection was envisaged as ‘an accessory to modern research’.^167^ Such research was always intended to cross the text–object boundary, because, as the museum's founding memorandum noted, ‘historic apparatus is so often illustrated in manuscripts and books.’^168^ And as a model made according to a manuscript description, Price's equatorium raises some important historiographical issues in this area, concerning the use that may be made of objects in interpreting texts, the relative value that may be attached to objects and texts by their collectors and, of course, why objects are produced from texts. This study has not addressed the Peterhouse manuscript itself, and many questions, not least that of authorship, have yet to be definitively answered. But the object produced from that manuscript has allowed us to take a fresh look at a hugely important period in both the history and the historiography of science.

## Notes

1 Although Price is best known by this name, he added the ‘de Solla’, his mother's maiden name, when he moved to the USA in 1957. The object is Wh.3271 (Whipple Museum of the History of Science, Cambridge).

2 ‘“The Equatorie of the Planetis”: exhibit at Royal Society Conversazione’, *The Times* (23 May 1952); ‘Ancient and modern’, *Birmingham Post* (23 May 1952).

3 See, for example, ‘Skrev Chaucer Bog om Astronomisk Regnemetode?’, *B.T*. (Copenhagen) (27 February 1952); ‘Possible Chaucer discovery’, *Auckland Star* (27 February 1952); ‘Was Chaucer a scientist too?’, *The Hindu* (Madras) (6 April 1952).

4 For details of what equatoria—and in particular this design—are and how they work, see Price's PhD thesis, published as *The Equatorie of the Planetis* (Cambridge University Press, 1955).

5 ‘Conversaziones 1952’, *Notes Rec. R. Soc. Lond*. **10**, 1–5 (1952), at pp. 3–4.

6 ‘Radio Newsreel’, *BBC Light Programme* [now BBC Radio 3], 1 March 1952; transcript courtesy of the Price family.

7 Eugene Garfield, ‘From the science of science to scientometrics: visualizing the history of science with *HistCite* software’, paper presented at 11th ISSI International Conference, Madrid, 25 June 2007 (see http://garfield.library.upenn.edu/papers/issispain2007.pdf, accessed 10 July 2013).

8 The Cavendish's role in mid-twentieth-century physics is discussed in Jeff Hughes, ‘Radioactivity and nuclear physics’, in *The Cambridge history of science* (ed. Mary Jo Nye), pp. 350–374 (Cambridge University Press). Further details of work at the Cavendish, and Bragg's restructuring and reform of it, are given below.

9 Exemplary in recent work on the material culture of science is Lorraine Daston (ed.), *Things that talk: object lessons from art and science* (Zone, New York, 2004).

10 Some scholars have documented their own production of replicas, to understand particular experiments or the function of certain instruments; notable in this regard is Heinz Otto Sibum, ‘Reworking the mechanical value of heat: instruments of precision and gestures of accuracy in early Victorian England’, *Stud. Hist. Phil. Sci.* **26**, 73–106 (1995). Sibum's work has been followed more recently by Roland Wittje and Hasok Chang (see, for example, H. Chang, ‘How historical experiments can improve scientific knowledge and science education: the cases of boiling water and electrochemistry’, *Science Educ.* **20**, 317–341 (2011); see also Frances Willmoth, ‘“Reconstruction” and interpreting written instructions: what making a seventeenth-century plane table revealed about the independence of readers’, *Stud. Hist. Phil. Sci.* **40**, 352–359 (2009).) However, I am of course interested in looking one level down from this, at the historiography of science. Perhaps the most discussed replica in the historiography of science is that produced by Thomas B. Settle (‘An experiment in the history of science’, *Science* **133**, 19–23 (1961)). Even this, however, has mostly been cited in discussions either of Galileo or of its potential re-reproduction in science education, rather than in its own right for its impact on our discipline. In any case, it was a replica experiment (albeit one that necessitated a certain amount of material reproduction) rather than an instrument. See also James MacLachlan, ‘Experimenting in the history of science’, *Isis* **89**, 90–92 (1998).

11 For the significance of Gunther's work and the 1936 exhibition, see J. A. Bennett, ‘The Cambridge Legacy of Robert T. Gunther’, in *Learning, language and invention: essays presented to Francis Maddison* (ed. W. D. Hackman and A. J. Turner), 78–83 (Variorum, Aldershot, 1994).

12 This period in the history of the discipline has been well assessed by J. A. Bennett, ‘Museums and the establishment of the history of science at Oxford and Cambridge’, *Br. J. Hist. Sci.* **30**, 29–46 (1997), and Anna-K. Mayer, ‘Setting up a discipline: conflicting agendas of the Cambridge History of Science Committee, 1936–1950’, *Stud. Hist. Phil. Sci*. A **31**, 665–689 (2000).

13 *Memorandum on the future of the history of science as a subject of study and research in the University with proposals for the creation of a University Museum and a University Department of the History of Science*, in ‘Documents from the early history of the museum’, compiled by Frances Willmoth, in *The Whipple Museum of the History of Science: instruments and interpretations, to celebrate the sixtieth anniversary of R. S. Whipple's gift to the University of Cambridge* (ed. Liba Taub and Frances Willmoth), pp. 11–55 (Cambridge University Press, 2006), at p. 13 (taken from Whipple Museum Papers C.54). For details of the memorandum's drafting, see Bennett, *op. cit.* (note 12), pp. 39–40.

14 The foundation of the British Society for the History of Science is discussed in Geoffrey Cantor, ‘Charles Singer and the early years of the British Society for the History of Science’, *Br. J. Hist. Sci.* **30**, 5–23 (1997). It has not been possible to dedicate much space to Singer in the present article, but it should be noted that he worked and corresponded with Price for some years; they had many historiographical interests (and some details of their personal backgrounds) in common.

15 J. Needham to C. Singer, 7 June 1948, Cambridge University Library (hereafter CUL), Needham Papers B.309.

16 C. Raven to J. Needham, 6 May 1948, CUL, Needham Papers B.309.

17 Imperial College, London, MS Hall (uncatalogued).

18 H. Butterfield to A. R. Hall, 6 February 1956, Imperial College, MS Hall. The first volume of *Science and civilisation in China* was published in 1954; Butterfield presumably objected both to its avowedly Marxist historiographical foundations and to the scientific background of its author, though it should be noted that Needham's work inspired him to take an academic interest in China himself (C. T. McIntire, *Herbert Butterfield: historian as dissenter* (Yale University Press, New Haven, CT, 2004), p. 337).

19 Herbert Butterfield, *The origins of modern science: 1300–1800* (G. Bell, London, 1949), p. ix.

20 *Idem*.

21 Alex Keller, ‘A collection to be preserved’, in Taub and Willmoth (eds), *op. cit.* (note 13), pp. 69–74, at p. 70.

22 CUL, Needham Papers B.308.

23 CUL, Needham Papers B.309, 27 May 1948.

24 A. R. Hall, ‘Whipple Museum of the History of Science, Cambridge’, *Nature* **167**, 878–879 (1951), at p. 879.

25 R. T. Gunther, *Early science in Cambridge* (Oxford University Press, 1937), p. v.

26 It was under Butterfield that the committee had ended the practice of inviting scholars from abroad to contribute to the history of science lecture series; Needham noted with regret the committee's reluctance to consider anyone from outside Cambridge for the post of curator and lecturer in the history of science in 1948. (J. Needham to C. Singer, 7 June 1948, CUL, Needham Papers B.309.) See Mayer, ‘Setting up a Discipline’, *op. cit.* (note 12), p. 681.

27 Joseph Needham and Walter Pagel (eds), *Background to modern science: ten lectures at Cambridge arranged by the History of Science Committee, 1936* (Cambridge University Press, 1938), p. viii.

28 *Memorandum*, *op. cit.* (note 13), p. 14.

29 The following section depends heavily on a wealth of information and evidence provided by Linda deSolla Price, Jeffrey Price and Mark de Solla Price.

30 Derek Price, Application for University of Cambridge, 23 February 1951, Cambridge University Archives Records of the Board of Graduate Studies (hereafter Cambridge UA BOGS) 1 1953–4, Price D. J. For the foundation and ethos of the South-West Essex Technical College, see ‘The South-West Essex Technical College’, *Nature* **145**, 775–776 (1940), and Harry Lowery, ‘A people's university’, *J. Higher Educ.* **18**, 37–39 (1947).

31 H. Lowery, reference for D. Price, 26 February 1951, Cambridge UA BOGS 1 1953–4, Price D. J.; D. Price, curriculum vitae 1959, Wellcome Collection PP/CJS/A.47.

32 D. Price, curriculum vitae 1956, Royal Institution MS WLB 55E/67.

33 Price, Cambridge application, *op. cit.* (note 30).

34 Derek Price, *Little science, big science … and beyond* (Columbia University Press, New York, 1986), p. xx. His eureka moment led to a paper, ‘Quantitative measures of the development of science’, at the sixth International Congress of the History of Science in 1950 (*Arch. Int. Hist. Sci.* **14**, 85–93 (1951)) and, arguably, to the field of scientometrics. It is for the establishment of a statistically based sociology of science, as well as his efforts to piece together the workings of the ‘Antikythera Mechanism’, that Price is now best known.

35 A. R. Hall to W. L. Bragg, 5 February 1951, Royal Institution MS WLB 59A/142; Linda deSolla Price, email correspondence with the author, 11 March 2013. Hall and Bragg were uneasy about Price's motives for giving up his post in Malaya, and took steps to investigate the situation (RI MS WLB 59A/142–145).

36 W. L. Bragg to S. Gorley Putt, 7 March 1951, Royal Institution MS WLB 59A/144.

37 W. L. Bragg to A. R. Hall, 19 May 1951, Imperial College, MS Hall.

38 H. Butterfield to W. J. Sartain, 26 February 1951, Cambridge UA BOGS 1 1953–4, Price D. J.; Hall to Bragg, 5 February 1951, Royal Institution MS WLB 59A/142.

39 Price, Cambridge application, *op. cit.* (note 30).

40 C. N. Parkinson, reference for D. Price, 28 February 1951, Cambridge UA BOGS 1 1953–4, Price D. J. As Raffles Professor of History in Malaya, Parkinson was a colleague of Price's.

41 T. H. Silcock, reference for D. Price, 1 March 1951, Cambridge UA BOGS 1 1953–4, Price D. J.

42 H. Lowery, reference for D. Price, 26 February 1951, Cambridge UA BOGS 1 1953–4, Price D. J.

43 W. L. Bragg to H. Lowery, 7 March 1951, Royal Institution MS WLB 59A/143.

44 W. L. Bragg to Civil Service Commission, 3 April 1959, Royal Institution MS WLB 55F/82.

45 W. L. Bragg to Nevill Mott, 4 May 1962, Royal Institution MS WLB 55F/89.

46 C. J. Singer to W. L. Bragg, 12 December 1955, Royal Institution MS WLB 55F/45. See also, for instance, C. J. Singer to J. F. Fulton, 27 March 1959, and to Edward Appleton, 21 November 1957, both Wellcome Collection PP/CJS/A.47.

47 W. L. Bragg to C. J. Singer, 15 December 1955, Royal Institution MS WLB 55F/47.

48 Snobbishness is certainly implicit in Nevill Mott's suggestion in 1962 that ‘Price has a chip on his shoulder in all matters concerning Cambridge because they did not appoint him to a lectureship’, but of course it is hard to gauge the relative weight of mutually reinforcing snobbishness and resentment from either side in that kind of comment (N. Mott to W. L. Bragg, 7 May 1952, Royal Institution MS WLB 55F/90).

49 Alan Mackay, ‘Derek John de Solla Price: an appreciation’, *Social Stud. Sci.* **14**, 315–320 (1984), at p. 315.

50 See, for example, Hall to Singer, 10 August and 13 September 1958, Wellcome Collection PP/CJS/A.42, and R. S. Whipple to Hall, 29 November 1952, Whipple Museum of the History of Science, Cambridge MS D108.

51 Rupert Hall to Marie Boas, 30 October 1956, Imperial College, MS Hall. I am most grateful to an anonymous referee for drawing my attention to this letter and the one referenced in the following note.

52 Marie Boas to Rupert Hall, 3 November 1956, Imperial College MS Hall.

53 W. L. Bragg to J. A. Ratcliffe, 4 March 1954, Royal Institution MS WLB 55F/6.

54 Jeffrey Price, email correspondence with the author, 8 March 2013; B. C. Griffith, ‘Derek Price (1922–1983) and the social studies of science’, *Scientometrics* **6**, 5–7 (1984), at p. 5.

55 *Cambridge University Reporter*, 22 January 1945, pp. 377–378. Fortunately for Price, a footnote to the Fellowship Regulations allowed the Fund managers to select Fellows for research in ‘any subject which, in the opinion of the Managers, is related to any of the branches of science enumerated in the deed of covenant.’ (p. 378n1).

56 Minutes of ICI Fellowships Fund meeting, 4 June 1951, CUL UA GB 501 Box 646.

57 *Cambridge Historical Register, Supplement 1951–55* (1956), p. 86; Minutes of ICI Fellowships Fund meeting, 9 June 1952, CUL UA GB 501 Box 646. The minutes note that Price's application for renewal was discussed ‘at length’; again it seems Bragg's support was invaluable.

58 *Cambridge Historical Register*, Supplement 1941–50 (Cambridge, 1952), p. 58; D. Price to W. J. Sartain, 8 May 1951 Cambridge UA BOGS 1 1953–4, Price D. J.

59 Minutes of History of Science Committee meeting, 14 November 1951, CUL, Needham Papers B.314.

60 Gunther, *op. cit.* (note 25), pp. 126–127.

61 Derek de Solla Price, *Science since Babylon*, enlarged edition (Yale University Press, New Haven, CT, 1975), pp. 26–27.

62 Jeffrey Price, email correspondence with the author, 5 March 2013.

63 ‘Possible Chaucer manuscript: discovery at Cambridge’, *The Times* (28 February 1952), p. 6; ‘Chaucer Holograph Found in Library’, *Varsity* (23 February 1952).

64 Derek Price, ‘The Equatorie of the Planetis’, *Times Lit. Suppl.* (2613) (29 February 1952), 164; and *Times Lit. Suppl.* (2614) (7 March 1952), 180.

65 R. S. Whipple to A. R. Hall, undated, in reply to Hall's letter of 24 December, Imperial College MS Hall.

66 Derek J. Price, ‘In quest of Chaucer—astronomer’, *Camb. Rev.* **76** (30 October), 123–124 (1954), at p. 123.

67 ‘Radio Newsreel’, *op. cit.* (note 6); Price, *op. cit.* (note 4), p. 165.

68 Derek Price, diary entry for 16 March 1952, courtesy of Jeffrey Price.

69 J. G. Crowther, *The Cavendish Laboratory, 1874–1974* (Macmillan, London, 1974) is, despite its analytical and stylistic flaws, still the most complete guide to the history of this institution.

70 David Phillips, ‘William Lawrence Bragg’, *Biogr. Mem. Fell. R. Soc.* **25**, 74–143 (1979), at p. 109; John Jenkin, *William and Lawrence Bragg, father and son: the most extraordinary collaboration in science* (Oxford University Press, 2008), p. 431.

71 John Kendrew, 1947, quoted in Graeme K. Hunter, *Light is a messenger: the life and science of William Lawrence Bragg* (Oxford University Press, 2004), p. 155.

72 Brian Pippard, ‘Bragg—the Cavendish Professor’, in *Selections and reflections: the legacy of Sir Lawrence Bragg* (ed. J. M. Thomas and D. C. Phillips), pp. 97–100 (Science Reviews, Northwood, 1990), at p. 98.

73 Lawrence Bragg, ‘Physicists after the war’, *Nature* **150**, 75–80 (1942).

74 *Ibid.*, p. 78.

75 Crowther, *op. cit.* (note 69), p. 276.

76 *Ibid.*, p. 287.

77 *Ibid.*, p. 267.

78 Lawrence Bragg, ‘Famous experimental apparatus in the Cavendish Laboratory, Cambridge’, *Nature* **166**, 7–9 (1950).

79 ‘Physics history’, *Daily Telegraph* (28 March 1952).

80 Derek Price, ‘The Cavendish Laboratory Archives’, *Notes Rec. R. Soc*. **10**, 139–147 (1953).

81 See Price correspondence within the Papers of James Clerk Maxwell, CUL Add.7655/IIIb/12,13.

82 Lawrence Bragg, ‘The Cavendish Laboratory Archives’, *Nature* **169**, 684–686 (1952), at p. 684.

83 CUL GBR/0265/CAV 20; Derek J. de Solla Price Papers, Adler Planetarium & Astronomy Museum, Chicago, Series 4, Box 2, Folder 50.

84 *Cambridge University Reporter*, *op. cit.* (note 55), p. 378.

85 Jeffrey Price, email correspondence with the author, 8 March 2013.

86 Malcolm Longair, conversation with the author, 18 February 2013.

87 D. J. Price, ‘Chaucer's astronomy’, *J. R. Instn* **35**, 396–407 (1952), at p. 405.

88 Price (ed.), *op. cit.* (note 4), p. 18.

89 *Ibid.*, p. 62.

90 Derek J. Price, ‘Precision instruments: to 1500’, in *A history of technology*, vol. 3 (*From the Renaissance to the Industrial Revolution, c. 1500–c. 1750*) (ed. Charles Singer *et al.*), pp. 582–619 (Clarendon Press, Oxford, 1957), at p. 584.

91 This was the headline the Birmingham Post used for their report of the evening: ‘Ancient and modern’, *op. cit.* (note 2).

92 ‘Conversaziones 1949’, *Notes Rec. R. Soc. Lond*. **7**, 54–60 (1949), at p. 54; for the origins of the Conversaziones, see ‘The Conversaziones of 1939’, *Notes Rec. R. Soc. Lond*. **2**, 127–139 (1939), and R. K. Bluhm, ‘A note on the origin of the Society's Conversaziones’, *Notes Rec. R. Soc. Lond*. **13**, 61–63 (1958).

93 Sally M. Horrocks, ‘The Royal Society, its Fellows and industrial R&D in the mid twentieth century’, *Notes Rec. R. Soc. Lond*. **64**, S31–S41 (2010).

94 Crowther, *op. cit.* (note 69), p. 269.

95 ‘Conversaziones 1952’, *op. cit.* (note 5), pp. 3–4.

96 Anthony Kelly, ‘Lawrence Bragg's interest in the deformation of metals and 1950–1953 in the Cavendish—a worm's-eye view’, *Acta Crystallogr.* A **69**, 16–24 (2013), at p. 22.

97 See, for example, ‘Conversaziones 1951’, *Notes Rec. R. Soc. Lond*. **9**, 138–143 (1951), at pp. 142–143; ‘Conversaziones 1953’, *Notes Rec. R. Soc. Lond*. **11**, 1–5 (1954), at p. 1.

98 Rupert Hall, ‘The first decade of the Whipple Museum’, in Taub and Willmoth (eds), *op. cit.* (note 13), pp. 57–68, at p. 65.

99 E. H. K. Dibden to A. R. Hall, 7 May 1952, Imperial College, MS Hall.

100 D. J. Price, undated [March or April 1952], handwritten note on Royal Society's ‘Notes for Exhibitors’, Imperial College MS Hall.

101 Derek Price, diary entry for 23 May 1952, courtesy of the Price family.

102 Price, *op. cit.* (note 4), p. 128.

103 Minutes of Merton College Governing Body meeting, 17 March 1952 (the instrument was insured for £500 for the duration of the loan); The Royal Society, *Conversazione, 22 May 1952* (unpublished programme), p. 12, courtesy of Jeffrey Price.

104 D. J. Price, ‘Exhibit No. 25’, unpublished notes from Royal Society Conversazione, 22 May 1952, courtesy of Jeffrey Price.

105 R. T. Gunther, *Early science in Oxford*, vol. 2 (*Astronomy*) (Oxford University Press, 1923), p. 208. It should be admitted that Gunther had only a limited understanding of equatoria (*ibid*., p. 234).

106 *Ibid.*, p. 2.

107 *Ibid.*, p. 208; Price, *op. cit.* (note 4), p. 129.

108 E. G. R. Taylor to A. R. Hall, 25 February 1952, Imperial College, MS Hall.

109 Price, *op. cit.* (note 87), p. 12.

110 Sir William Osler, ‘The Old Humanities and the New Science: the Presidential Address delivered before the Classical Association at Oxford, May, 1919’, *Br. Med. J.* **ii**, 1–7 (1919); R. T. Gunther, *Early science in Oxford*, vol. 1 (*Chemistry, mathematics, physics and surveying*) (Oxford University Press, 1923), p. v.

111 See, for example, *The Times*, *op. cit.* (note 2); ‘Royal Society Conversazione’, *Pharmaceut. J.* (31 May 1952); ‘Science in Piccadilly’, *Br. Med. J.* **i**, 1182–1183 (1952).

112 Price, *op. cit.* (note 87).

113 Hall, *op. cit.* (note 98), p. 65.

114 R. S. Whipple to A. R. Hall, 29 November 1952, Whipple Museum Papers D.108.

115 A. R. Hall, Report on D. J. Price (undated, *ca.* Easter 1952), Cambridge UA BOGS 1 1953–4, Price D. J.

116 D. J. Price to L. M. Harvey, and R. F. Bennett to L. M. Harvey, 4 and 9 May 1952, Cambridge UA BOGS 1 1953–4, Price D. J.

117 This unusual situation arose after some negotiation in which a range of alternative suggestions were proposed and rejected in turn. These included waiving the requirement for an oral examination, having only one oral examiner, and examining Price in writing. In the end Price's oral examination took place on 14 May 1954; the other examiner was E. G. R. Taylor. (F. H. Hinsley to L. M. Harvey, 11 February 1954; L. M. Harvey, record of telephone conversation with F. H. Hinsley, 17 February 1954; E. G. R. Taylor and A. R. Hall, ‘Examiners’ reports on a dissertation submitted for the Ph.D. degree entitled ‘The Equatorie of the Planetis’ by D. J. Price of Christ's College'; all in Cambridge UA BOGS 1 1953–4, Price D. J.)

118 Minutes of ICI Fellowships Fund meeting, 9 June 1952, CUL UA GB 501 Box 646.

119 *Idem*.

120 Derek J. Price, ‘The Equatorie of the Planetis’, *J. South-West Essex Tech. Coll. Sch. Art* **3**, 154–169 (1952), at p. 169.

121 Keller, *op. cit.* (note 21), p. 71.

122 Maurice Daumas, *Les Instruments Scientifiques Aux XVIIe et XVIIIe Siècles* (Presses Universitaires de France, Paris, 1953), p. 9.

123 *Ibid.*, p. 1.

124 Hall, *op. cit.* (note 98), pp. 62–63.

125 Frank James, ‘Alfred Rupert Hall and Marie Boas Hall’, *Biogr. Mems Fell. Br. Acad.* **11**, 353–408 (2012), at p. 364. For discussion of the influence of Sarton, and comparison with Thorndike and Koyré, see Arnold Thackray and Robert K. Merton, ‘On discipline building: the paradoxes of George Sarton’, *Isis* **63**, 473–495 (1972).

126 Rupert Hall, ‘The scholar and the craftsman in the Scientific Revolution’, in *Critical problems in the history of science* (ed. Marshall Clagett), pp. 3–23 (University of Wisconsin Press, Madison, WI, 1959), at p. 18.

127 Scott Mandelbrote, ‘A. Rupert Hall’, *Metascience* **5**, 64–84 (1994), at p. 67 (presenting edited excerpts of a long interview; the full transcript is in the archives of All Souls College, Oxford). It should be noted that Hall came to criticize the knee-jerk rejection of progress-focused ‘Whig’ history which was so strongly associated with Butterfield; see Hall, ‘On Whiggism’, *Hist. Sci.* **21**, 45–59 (1983).

128 Hall to Butterfield, 28 August 1951, Imperial College, MS Hall.

129 Brendan Simms, ‘Butterfield, Sir Herbert (1900–1979)’, *Oxford dictionary of national biography* (Oxford University Press, 2004) (see http://www.oxforddnb.com/view/article/30888, accessed 13 May 2013).

130 A. R. Hall and M. B. Hall, *The correspondence of Henry Oldenburg* (13 volumes) (University of Wisconsin Press, Madison, WI, 1965–86). See James, *op. cit.* (note 125), especially pp. 395–398.

131 Rupert Hall, ‘Beginnings in Cambridge’, *Isis* **75**, 22–25 (1984), at p. 24.

132 Hall, *op. cit.* (note 126), pp. 4–5.

133 Price (ed.), *op. cit.* (note 4), p. xiii.

134 F. Sherwood Taylor, ‘Museum of the History of Science, Oxford’, *Nature* **164**, 738–739 (1949), at p. 739. Taylor had fought on the western front in World War I (Frank Greenaway, ‘Taylor, Frank Sherwood (1897–1956)’, *Oxford dictionary of national biography* (Oxford University Press, 2004) (see http://www.oxforddnb.com/view/article/36430, accessed 18 September 2013)).

135 Hall, *op. cit.* (note 98), pp. 62–63.

136 J. T. Saunders to H. C. Marshall, 12 May 1950, Whipple Museum Papers D.27.

137 See, for example, minutes from 14 November 1951; Report to General Board, May 1952, CUL, Needham Papers B.314/315.

138 J. Needham to E. Wynn, 9 October 1953, Whipple Museum Papers D.140; Minutes of History of Science Committee, 14 June 1954, CUL Needham Papers B.319.

139 A. R. Hall, memorandum to History of Science Committee, considered 29 November 1954, CUL, Needham Papers B.320.

140 E. D. Barlow to J. Needham, 18 February 1969, CUL Needham Papers B.346.

141 Bennett, *op. cit.* (note 12), p. 44.

142 A. R. Hall, memoranda to History of Science Committee, 29 November 1954 and 9 November 1956, CUL Needham Papers B.320 and 325.

143 Hoskin was appointed Lecturer in History of Science in 1959, in succession to Hall, without an interview (Michael Hoskin, email correspondence with the author, 27 June 2013). This caused ‘traumatic shock’ to Price, the other candidate for the post (D. J. Price to C. J. Singer, 20 July 1959, Wellcome Collection PP/CJS/A.47).

144 David Bryden, email correspondence with the author, 18 October 2012.

145 Minutes of Committee of Management for Biophysical Chemistry and Colloid Science, 27 January 1970, Cambridge University Library CSPS 8/6; memorandum on future accommodation for colloid science, 2 May 1966, CUL Roughton B.42.

146 David Bryden, email correspondence with the author, 18 October 2012; see also Bryden's correspondence with V. M. Harries about the equatorium, 2 April and 8 April 1975, Whipple Museum Papers 561A/B.

147 Whipple Museum of the History of Science, accessions register for Wh.3271. Wh.3270, accessioned at the same time, was a Ramsden dividing engine that had also only just made it back from the Thompson's Lane store.

148 Wh.3271 catalogue entry as at 16 October 2012. The duty of museum curators to give pithy names to objects is nothing new, but databases have had a sometimes unexpected impact. For discussion of these issues, see Ross Parry (ed.), *Museums in a digital age* (Routledge, Abingdon, 2010).

149 Credit for the rediscovery must go to Claire Wallace and Steve Kruse at the Whipple Museum: their comprehensive knowledge of the collection allowed my vague inquiries to meet with precise results.

150 See Sherwood Taylor, *op. cit.* (note 134), p. 739.

151 Gunther, *op. cit.* (note 25), p. v.

152 Hall, *op. cit.* (note 24), p. 879.

153 D. Bryden, email correspondence with the author, 18 October 2012.

154 Whipple Museum of the History of Science, Cambridge: Wh.0424, Wh.0425, Wh.0975, Wh.1817 and Wh.1818.

155 Whipple Museum of the History of Science, Cambridge: Wh.2662.

156 They were displayed with pride as replicas even before the museum opened in 1951. See University of Cambridge History of Science Lectures Committee, *A guide to the historic scientific instruments in the Whipple Museum of the History of Science* (1949), p. 19.

157 Price came close to admitting this in his Royal Institution Discourse (Price, *op. cit.* (note 87), p. 7).

158 D. J. de Solla Price to C. J. Singer, 17 October 1959, Wellcome Collection PP/CJS/A.47.

159 Malcolm S. Kirk, email correspondence with the author, 29 May 2013.

160 Anthony Liversidge, ‘Why Einstein is overrated: interview: Derek de Solla Price’, *Omni* (December), 89–102 and 136 (1982), at p. 89.

161 The maker, not mentioned in the *Omni* article, was Bob J. Deroski of Long Island (email correspondence with Linda deSolla Price, 20 June 2013). Cf. Steven Shapin, ‘The invisible technician’, *Am. Scient.* **77**, 554–563 (1989).

162 Price, *op. cit.* (note 61), p. xvi.

163 J. A. Ratcliffe, quoted in Phillips, *op. cit.* (note 70), p. 123.

164 Sir Lawrence Bragg, ‘Foreword’, in James D. Watson, *The double helix: a personal account of the discovery of the structure of DNA* (Weidenfeld, London, 1968), p. ix.

165 British Crystallographic Association, ‘Bragg Centenary’ (see http://crystallography.org.uk/bragg-centenary/, accessed 16 July 2013); University of Leeds, ‘Bragg Centenary 2013’ (see http://www.leeds.ac.uk/info/125160/bragg_centenary_2013, accessed 16 July 2013).

166 C. J. Singer to Sir Edward Appleton, 21 November 1957, Wellcome Collection PP/CJS/A.47. Singer and Hall had worked closely together, including on *A history of technology* (quoted above). Singer examined Hall's PhD, and it is clear that there was mutual professional respect (James, *op. cit.* (note 125), p. 363). So we should perhaps not read too much into Singer's remarks.

167 *Memorandum*, *op. cit.* (note 13), p. 14.

168 *Ibid.*, p. 16; for a discussion of texts and objects in the context of the Whipple Museum, see Adam Mosley, ‘Spheres and texts on spheres: the book–instrument relationship and an armillary sphere in the Whipple Museum of the History of Science’, in Taub and Willmoth (eds), *op. cit.* (note 13), pp. 301–318.

